# Effect of Post-Cured through Thickness Reinforcement on Disbonding Behavior in Skin–Stringer Configuration

**DOI:** 10.3390/ma17143389

**Published:** 2024-07-09

**Authors:** Jimesh D. Bhagatji, Christopher Morris, Yogaraja Sridhar, Bodhisatwa Bhattacharjee, Krishnanand N. Kaipa, Oleksandr G. Kravchenko

**Affiliations:** Department of Mechanical and Aerospace Engineering, Old Dominion University, Norfolk, VA 23529, USA; cmorr023@odu.edu (C.M.); kkaipa@odu.edu (K.N.K.)

**Keywords:** through-thickness reinforcement, crack resistance, interlaminate toughing, cohesive zone modeling

## Abstract

An experimental investigation of interlaminar toughness for post-cured through-thickness reinforcement (PTTR) skin–stringer sub-element is presented. The improvement in the crack resistance capability of skin–stringer samples was shown through experimental testing and finite element analysis (FEA) modeling. The performance of PTTR was evaluated on a pristine and initial-disbond of the skin–stringer specimen. A macro-scale pin–spring modeling approach was employed in FEA using a non-linear spring to capture the pin failure under the mixed-mode load. The experimental results showed a 15.5% and 20.9% increase in strength for the pristine-PTTR and initial-disbond PTTR specimens, respectively. The modeling approach accurately represents the overall structural response of PTTR laminate, including stiffness, adhesive strength, crack extension scenarios and progressive pin failure modes. This modeling approach can be beneficial for designing damage-tolerant structures by exploring various PTTR arrangements for achieving improved structural responses.

## 1. Introduction

Stiffened composite structures [1] have become increasingly prevalent in the load-bearing sections of modern aircraft airframes due to their efficient load distribution and significant weight reduction [2]. These structures consist of a composite skin reinforced by stiffening elements such as stringers used for various load-carrying conditions in aircraft. Co-bonding [3] and secondary adhesive bonding [4,5,6] have become the preferred modes for composite component joining methods as an alternative to riveting for the airframes, as these prevent reduced notch strength [7,8] and are lightweight compared to fastening. However, the use of adhesive-stiffened composite structures is sensitive to manufacturing defects, free edge stresses [9,10,11] and impact events [12], leading to interlaminar [13] fracture or skin–stringer disbonding, along with intralaminar damage like matrix cracking and fiber failure. However, the need for effective crack-arresting reinforcements has become a necessity with the increase in large, complex airframe structures [14,15].

The major limitation in traditional laminated composites and adhesive bonded composites is due to weak interlaminar toughness, which is primarily due to the absence of through-thickness fiber reinforcement [16]. One of the high-performance through-thickness reinforcement (TTR) techniques used in industry practices involves dry fabric preforms where through-thickness fibers are incorporated during the fabric production [17], prior to resin infusion. Examples include 3D weaving, stitching, or braiding techniques. Another technique is Z-pinning [18], where uncured prepreg laminates are reinforced in the through-thickness direction by inserting reinforcing pins. Both approaches effectively create a three-dimensional (3D) composite material with a resulting increase in resistance to delamination propagation. Incorporating these Z-fibers disturbs the in-plane fiber orientation, producing undesired features like resin-rich regions around Z-pinning fibers [19] and Z-fiber stitching patches in fabric preforms [20], which cause a knockdown in in-plane mechanical performance. An ideal TTR technique aims to reduce the in-plane fiber damage and associated in-plane mechanical performance while increasing interlaminar toughness. Recently, a new TTR technique has been explored where small, sub-millimeter holes are drilled orthogonally in post-cured laminates, and then filled with low-viscosity resin, followed by inserting the carbon fiber rods into the holes providing for the bonding to the parent laminate once the infused resin is cured [21]. The potential of the post-cure through-thickness reinforcement (PTTR) technique is to reduce its effect on the in-plane fibers by ensuring that the hole’s center-to-center distance is relatively high [22,23] and the notch diameter is small, thus taking advantage of the notch-size effect [24]. PTTR can also help maintain pin orientation, unlike Z-pinning, where Z-fibers are oriented before consolidation, resulting in undesired pin alignment [25]. Finally, a significant advantage PTTR contributes over traditional TTR is in surface patch repair [26] by regaining the structural stiffness and maintaining structural integrity.

The preliminary work on PTTR emphasized on bridging the force of pin pull-out under mode I loading [21] and the effect of pin aspect ratio on mode I fracture toughness [27,28]. The current work investigates the resistance to disbonding of PTTR for a skin–stringer configuration. To simulate the stringer disbonding loading condition experienced by aircraft crown fuselage panels [29], a simplified skin–stringer coupon [30] under three-point bending was used. The mechanical performance of PTTR was investigated for adhesive bond skin–stringer coupon for two scenarios: (i) pristine bondline with PTTR; and (ii) specimen with an initial, 6 mm (a_0_), disbond in the bondline with PTTR. These scenarios were compared against a baseline of an adhesively bonded stringer without PTTR. The proposed FEA modeling approach of the skin–stringer configuration considered the presence of the pin by explicitly modeling the holes within the parent laminate and treating the pin as a non-linear spring. This pin–spring modeling approach was used to analyze different pin failure modes, such as pin rapture and pin pull-out, expanding the modeling capabilities of TTR composite laminates compared to previous studies [31]. This research aimed to demonstrate the performance of the PTTR technique in enhancing the structural integrity of adhesive bonds in stiffened composite structures. Additionally, the proposed computational approach can be used in the design, development and validation of PTTR stiffened composite structures.

## 2. Skin–Stringer Specimen Fabrication and Mechanical Test

### 2.1. Skin–Stringer Specimen Fabrication

**Figure 1:**
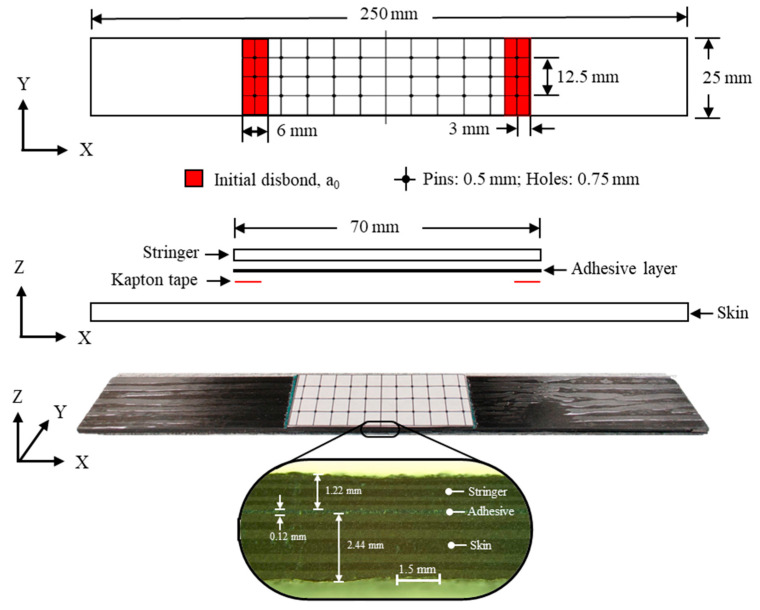
Skin–stringer specimen.

The skin–stringer sample configuration in this work adopted the previously validated sample geometry used by NASA as a part of the verification and validation process plan [32] based on developed coupon, sub-element, element, and sub-component configurations. The smallest sub-element test configuration in the validation block is a three-point bending skin–stringer specimen (shown in Figure 1). In this study, two types of skin–stringer specimens were prepared: one with a pristine and another with an initial-disbond (shown in Figure 1).

The skin lay-up comprised 16 plies, [0_2_/90_2_]_2s_, while the stringer lay-up consisted of eight plies, [0/90]_2s_. Both the skin and stringer were constructed from carbon fiber IM7/8552 epoxy prepreg tape, with a ply thickness of 0.15 mm. Specimen preparation began with laying up and vacuum bagging the skin and stringer ply layup panels, sized for a batch of eight specimens, and curing them separately in a Wabash Heat Press, following a two-stage cure process. The first stage involved heating to 90 °C at 25 psi pressure for 60 min, followed by a second stage at 170 °C at 80 psi for another 120 min under vacuum at 25 inHg. Subsequently, surface preparation was performed using 120 grit paper. The pristine specimen was fabricated by adhesively bonding the stringer to the skin using Henkel EA 9696 AERO epoxy film, sealing it in a vacuum bag, and curing it at 120 °C and 25 psi for 90 min in an autoclave. During curing, the adhesive flowed outward, resulting in an average bondline thickness of 0.12 mm. The bonded skin–stringer panel was then cut to the dimensions shown in Figure 1 using a waterjet to obtain the pristine specimen. Similarly, the initial disbond specimen was prepared, where skin and stringer panels were prepared separately in a heat press, but a 6 mm Kapton tape was inserted at the ends of the stringer under the adhesive film to simulate the initial disbond, as illustrated in Figure 1.

The TTR specimens were prepared by marking and drilling a 3 × 5 array of 0.75 mm holes, spaced 6.25 × 6 mm apart, using a Dremel with a diamond-plated drill bit tip. This parameter was selected by previous studies [27,28] to maintain an aspect ratio (AR) of 2.42. Subsequently, each hole was inspected under a microscope. Next, 0.5 mm pultruded unidirectional carbon fiber rods from Rock West Composites were dipped in INF-211/INF-114 epoxy resin, and wetted pins were inserted into the holes. The TTR specimens were left to cure at room temperature for 12 h or more.

### 2.2. Mechanical Testing

**Figure 2:**
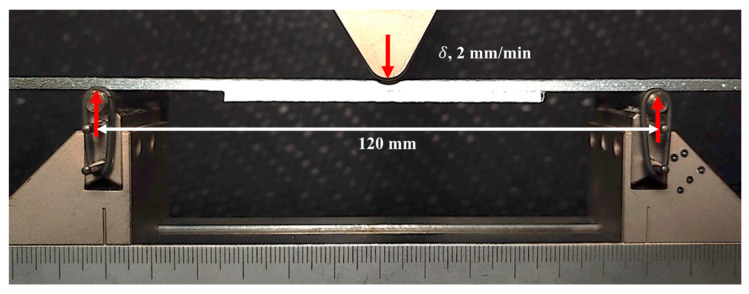
Three-point bending experimental setup for skin–stringer specimen.

To ensure relevance to real-world aerospace applications, three-point bending loading was selected to study PTTR bondline failure progression under controlled quasi-static conditions. While real-world disbonding often occurs under fatigue loading, the failure progression is expected to be similar. This approach helps characterize initial responses and failure mechanisms, with further testing under fatigue and additional loading conditions needed for full validation and optimization. Under monotonic three-point bending (Figure 2), the skin–stringer bondline experiences mixed-mode loading, where shear load or mode II is initiated due to shear deformation at the edge of the joint, and opening mode I bridging force is created due to peel stresses at the free-edge [33]. The fixed bottom roller was placed at 120 mm with the stringer facing down, and the top roller applied a displacement rate of 2 mm/min. To accurately represent the skin–stringer interface disbond/crack propagation, the GOM 3D digital image correlation (DIC) setup was used.

## 3. FEA Modeling Strategy

### 3.1. Pin Representation Based on Non-Linear Spring Model

The experimental results were investigated in combination with progressive failure modeling, which considered the effect of the drilled holes, and modeled each pin as a discrete non-linear spring with the effective properties of PTTR. While some similarities can be drawn from the Z-pinning modeling approach as a starting point, numerous modeling methods have been proposed to describe the material–structural response of z-pinned laminates. These include analytical [34], semi-analytical [31], and numerical [35], [36,37] approaches utilizing the finite element method. However, developing a generalized analytical model for sub-elements like skin–stringer specimens poses challenges. Numerical modeling is favored to capture complex large-scale bridging effects [36], but depending on the fidelity, computational modeling where the progressive damages pin can be modeled along with an interlaminar cohesive zone between skin–stringer may lead to a complex micro-mechanics model [35]. This has led to the development of various modeling approaches like the smeared cohesive model [31] and unit strip cohesive pin model [36] to avoid the modeling of a pin, thereby eliminating mesh-size dependency and enabling more accurate computational solutions. While these methods can incorporate large-scale bridging effects to determine structural responses, they may fail to depict the impact of TTR on in-plane structural response. Consequently, a new modeling strategy is proposed to represent the large-scale bridging effect of TTR rods using a spring–pin model [21] inspired by an analytical approach [34].

### 3.2. Cohesive Zone Modeling (CZM) of Skin–Stringer Interface

**Figure 3:**
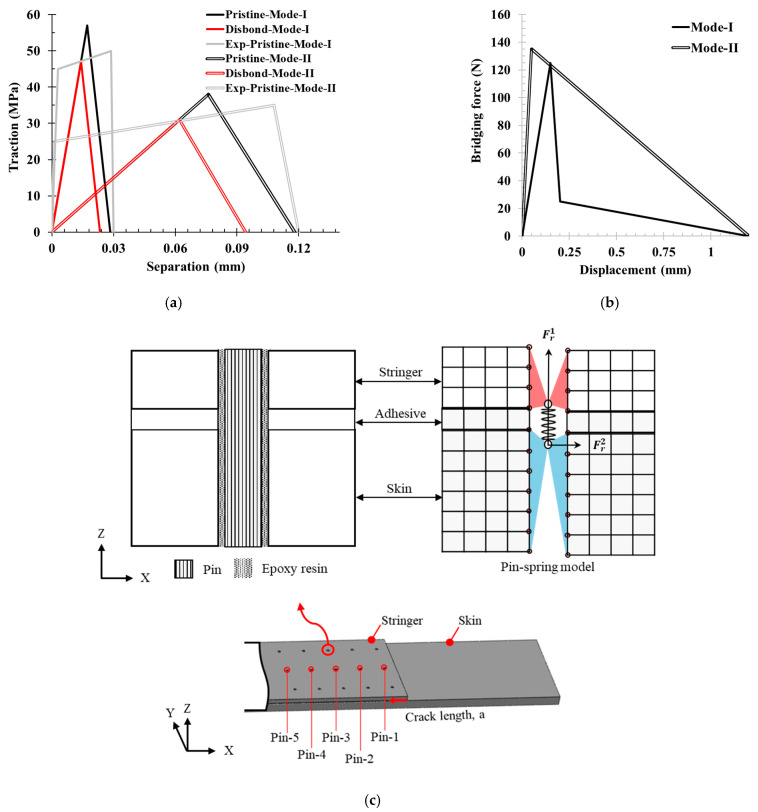
(**a**) Comparation of bilinear traction–separation for CZM of the pristine and disbond adhesive layer with experimental trapezoidal traction-separation behavior [38] (**b**) Bridging force–displacement behavior of single pin under pure mode I and mode II loading. (**c**) FEA model representation of a spring–pin model.

A full-scale skin–stringer model of a sample was constructed using the Abaqus standard (version 2023) to capture quasi-static bending. Eight-node linear continuum shell elements with reduced integration (SC8R) were used for the skin–stringer laminates. The mechanical properties of the laminate are shown in Table 1. A single layer of eight-node cohesive elements (COH3D8) was used for the bonding interface. The fracture toughness of the bonded joints was governed by a bilinear traction–separation law for further simplification of the mixed-mode behavior. To accurately predict delamination propagation, the pure mode (mode I and mode II) of traction–separation behavior of Henkel EA 9696 was determined from experimental results of lap joint and butt joint tests from ref. [38], as shown in Figure 3a. The effective penalty stiffnesses (*K_i_*) were determined from the ratio of interfacial strength (*T*, *S_ij_*) to critical separation (Δ*_c_*) to approximate the trapezoidal traction–separation behavior. The critical fracture energies ($G_{i}^{C}$) were determined considering normal and shear separation at failure (Δ*_f_*) determined from Ref. [38]. In this study, the shear strengths in the two orthogonal directions, *S*_1_ and *S*_2_, are assumed to be equal. Traction–separation parameters were separately determined for pristine and initial-disbond specimens and further validated with experimental testing, as shown in Figure 3a. Under bending, the bondline experienced mixed-mode loads; a quadratic stress criterion, shown in Equation (1), was used as the damage initiation criterion of the cohesive element, which triggers a softening response of the cohesive element, shown in Equation (2), using damage variable (*d*). The damage variable ranges from *d* = 0 in the elastic region of the traction separation plot to *d* = 1 at the end of the softening. The mixed-mode damage response ($d^{m}$) is captured by damage evolution criterion; an energy-based Benzeggagh and Kenane (B-K) condition [39] shown in Equation (3) was used, where *η* is the B-K mixed-mode factor, *G_S_*/*G_D_* is mixed-mode ratio, effective traction at damage initiation ($\sigma^{m})$ and maximum effective displacement (${∆}_{max}^{m})$ was determined by the numerical model (Equation (4)).

(1) $${\left(\frac{\sigma_{1}}{T}\right)}^{2}+{\left(\frac{\tau_{12}}{S_{12}}\right)}^{2}+{\left(\frac{\tau_{13}}{S_{13}}\right)}^{2}=1$$

(2) $$\sigma_{i\;}=\left(1-d\right)K_{i}\Delta_{i},\;0\leq d\leq 1,\;i=1,2,3$$

(3) $$G_{C}=G_{I}^{C}+\left(G_{II}^{C}-G_{I}^{C}\right)\;{\left(\frac{G_{S\;}}{G_{D}}\right)}^{\eta },\;G_{S\;}=G_{II}+G_{III},\;G_{D\;}={G_{I\;}+G}_{II}+G_{III}$$

(4) $$d^{m}=\frac{{∆}_{f}^{m}\;\left({∆}_{max}^{m}-{∆}_{c}^{m}\right)}{{∆}_{max}^{m}\;\left({∆}_{f}^{m}-{∆}_{c}^{m}\right)},\;{∆}_{f}^{m}=\frac{2G_{C}}{\sigma^{m}}$$

where $\sigma_{i\;}$ is the softening traction after critical separation of cohesive element and indexes 1, 2, and 3 represent in opening mode (mode I), sliding mode (mode II) and tearing mode (mode III), respectively. ${∆}_{c}^{m}\;$ and ${∆}_{f}^{m}$ is critical traction separation and separation at failure in mixed mode.

As the cohesive elements are sensitive to element size in the direction of crack propagation direction, a maximum critical element size of 0.33 mm was determined based on guidelines suggested in [40,41]. The rollers of the three-point bending setup are modeled using discrete rigid shell elements with finite sliding and contact interaction using a penalty-based tangential and normal hard contact. The bottom two rollers are fixed, while a 9 mm linear ramp displacement is given to the top roller. A mesh size of 0.33 mm is applied for the laminate continuum shell elements in the stringer region and 1 mm in the remaining region.

### 3.3. Representation of PTTR Using Discrete Pin–Spring Model in Skin–Stringer Configuration

The single-pin model is shown in Figure 3. A 3D non-linear spring element (CONN3D2) was used to simulate the bridging effect of the pins. At the pin mounting location, a through hole was modeled across the skin and stringer (as shown in Figure 3c) with two reference points: one coupled to the nodes at the skin side hole and another point coupled to the nodes at the stringer side hole. These reference points act as contact points for the spring element to simulate the pin bridging effect, using a force–separation constitutive law. The 3D spring element coupled with skin–stringer side node holes captures the effect of the hole being filled with resin and pin. Depending on skin–stringer–pin samples’ dimension configuration and mode I/mode II loading ratio, the pin failure mode can change. Furthermore, pin failure is highly dependent on the AR for PTTR [27], resulting in increased interlaminar strength as AR increases for mode I. Based on the current stringer thickness and pin diameter, AR = 2.42, pin pullout behavior was assumed; thus, a trilinear force–separation bridging law was considered, as shown in Figure 3b. This bridging law captured three phases of pin pullout. First, an elastic response was considered where a perfect bond was assumed between the pin, resin, and laminate hole. In the second phase, an instantaneous drop in the bridging force occurs due to the pin disbonding as a result of interface failure between the pin–epoxy or epoxy–laminate hole. In the last phase, through-thickness pin slip-outs under interfacial friction lead to a complete pin pullout.

For mode II, given the skin–stringer configuration, shear pin pullout was assumed instead of rupture [36]; thus, a bilinear force–separation bridging law was considered, as depicted in Figure 3b. The first phase represented the elastic shear stiffness of the pin. Once the pin reached the shear strength and disbonded, the second, softening, phase was initiated, with progressive force degradation resulting from large-scale bridging until complete pullout. In both bridging laws for pure modes, the failure separation was assumed to be equal to the thickness of the stringer; as the stringer is half the size of the skin, the shear resistance of the pin on the stringer side will be lower compared to the skin side. With this assumption, a displacement-controlled pin–spring failure criterion was implemented without considering the pin mixed-mode coupling, wherein the spring was deactivated in all the modes if any mode displacement exceeded the failure displacement for the respective mode. This maximum displacement criterion was used to capture different pin failure modes, including pin pullout or shear failure under large-scale bridging of skin–stringer configuration.

### 3.4. CZM Validation

**Figure 4:**
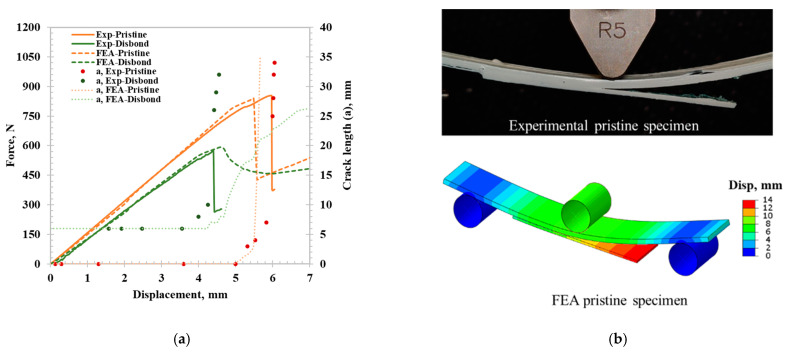
Skin–stringer CZM validation results: (**a**) force-displacement curve and crack length comparison between experimental and FE model for pristine and initial disbond specimens. (**b**) Post adhesive disbonding for pristine specimen.

Before analyzing the performance of PTTR using the spring–pin model, it is important to validate the CZM for the adhesive disbonding behaviors of the pristine and initial disbond specimen. As shown in Figure 4a, the model accurately captures the mechanical response of the skin–stinger sample, namely the initial stiffness and strength of 854.66 N. The crack initiation is captured when deviation from linear behavior is observed, leading to sample failure. The CZM captured rapid delamination propagation, which was also observed in the experiment, as shown in Figure 4b. Similarly, a close agreement was observed in the reduced stiffness and strength of the sample with the initial disbond. However, instead of the sudden delamination, which was observed experimentally, the CZM predicted a more gradual delamination growth.

## 4. PTTR Skin–Stringer Disbonding Analysis

**Figure 5:**
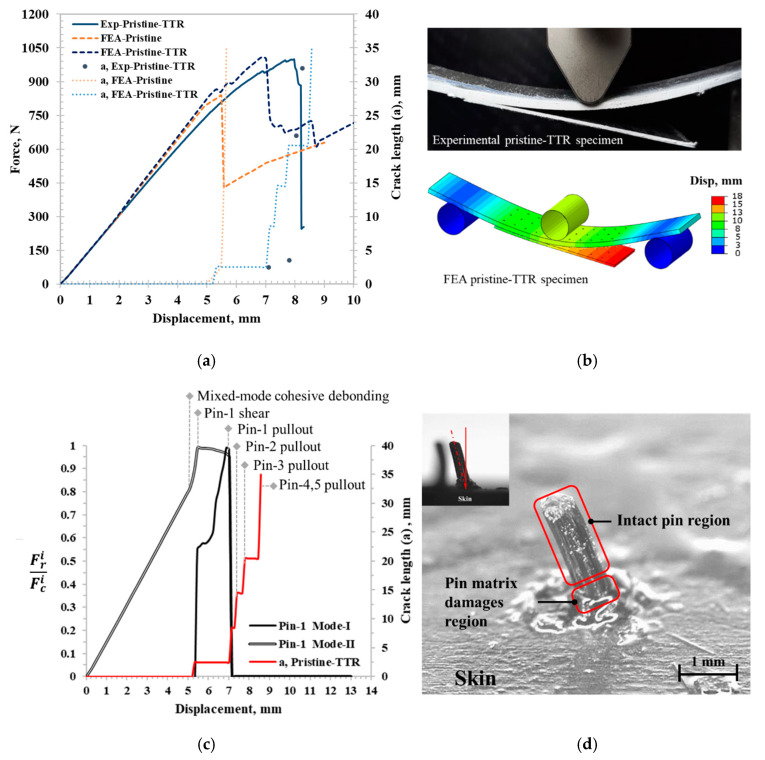
(**a**) Force–displacement curve and crack length comparison between experimental and FE model for the pristine-TTR specimen. (**b**) Post adhesive disbonding for pristine-TTR specimen. (**c**) Pin failure analysis using FE model: spring reaction force fraction vs. displacement for pristine-TTR under bending. (**d**) Post-pin-failure microscopy analysis.

The load–displacement curves and crack length for the experimental bending test and FE model results of the pristine-TTR specimen are presented in Figure 5a. The FE models accurately captured all distinct phases observed in the experimental test (shown in Figure 5b). The pristine-TTR FE model had a stiffness of 167.4 N/mm which compared closely to experimentally observed stiffness. The experimental crack growth of pristine-TTR specimen initiated with adhesive disbonding at a displacement 7 mm, which is a 41% increase in crack initiation displacement compared to pristine specimen. The adhesive disbonding in FEA of pristine-TTR was observed at close to 5 mm displacement, compared to 7 mm during the experiment, which is likely due to missing plastic behavior in bilinear CZM of the adhesive used in the FE modeling approach, which only considers elastic and fracture behavior. The FE model effectively represents the crack resting capability of the PTTR, starting with first-row springs at 3 mm. Following the crack arrest, the force increased until the failure strength of 1009.77 N, very close to the experimental failure strength, which is 15.5% higher compared to pristine. This sample configuration resulted in TTR with AR of 2.42; however, skin–stringer thickness in aerospace application can be higher than what was used in this study, resulting in an AR of 6–8 for same TTR pin diameter [42], which can increase the PTTR performance by 2 to 4 times [27]. In the experimental specimen, all pins failed instantly, resulting in a sudden drop in force at a displacement of 8 mm. Where else, the FE model exhibited similar instantaneous spring failure in the first row of springs and progressive failure in the next rows of springs. Experimentally, the pin may be misaligned (±3.94°) as the hole diameter was slightly larger than the pin, which can also contribute to the discrepancy in the pin failure modes. Further spring failure analysis was performed to determine the spring failure sequence shown in Figure 5c using the spring reaction force fraction ($F_{p}^{i}$), a ratio of spring reaction force from the FE model ($F_{r}^{i}$) to pin critical failure force ($F_{c}^{i}$) of respective modes, shown in Equation (5).

(5) $$F_{p}^{i}=\frac{F_{r}^{i}\;}{F_{c}^{i}\;},\;i=1,2$$

where 1 is in mode I direction and 2 is in mode II direction, as shown in Figure 3c.

Further, spring failure analysis was performed on pin 1, the middle of the first row, as shown in Figure 5c. With the initiation of cohesive disbonding, the shear force in the spring rapidly increased, leading to pin matrix damage at the skin–stringer interface, resulting in local spring shear failure (mode II). Following shear failure, the normal force surged in pin 1, leading to spring failure due to mode I pullout. It is evident from Figure 5c that pin shear failure occurred prior to mode I pin pullout. Since spring mode I pullout occurred immediately after spring shear failure, pin matrix failure remained localized, and the rest of the pin remained intact, as shown in Figure 5d. Upon pin 1 failure, the crack extended to the second spring row, which increased the mode I load, ultimately resulting in spring pin 2 pullout, followed by pins’ 3, 4, and 5 pullouts.

**Figure 6:**
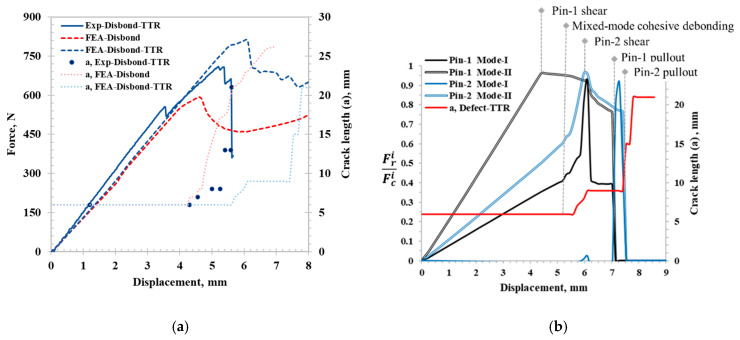
(**a**) Force–displacement curve and crack length comparison between experimental and FE model for the disbond-TTR specimen. (**b**) Pin failure analysis using FE model: spring reaction force fraction vs. displacement for disbond-TTR under bending.

Similarly, the load–displacement and crack length analyses were performed on the initial disbond specimen between the experimental bending test and FE model results, as shown in Figure 6a. The stiffness of the disbond-TTR FE model closely matched the experimental results. Initially, the experimental disbond-TTR results exhibited high stiffness because the stringer edge bonded to the skin outside the disbond region during the pin insertion. Hence, the stringer edge got disbonded at a lower load, which did not affect the initial disbonded crack length. The experimental failure strength was 682.15 N, which was a 20.9% increase compared to the specimens with the initial disbond. The FE model captured the region of extended stable crack propagation; however, it over-predicted the failure strength by 18.9%. In the experimental TTR specimens before the ultimate failure load, the adhesive layer was completely disbonded and the stringer was still held by TTR pins, making it challenging to determine adhesive delamination in the experimental tests. Therefore, the crack propagation in TTR coupons was tracked by considering the surface separation of the crack rather than the failure of cohesive elements. Experimental crack growth in the disbond-TTR specimen began with adhesive disbonding at the crosshead displacement of 4.25 mm, followed by crack arrest at the second and third rows of pins, as shown in Figure 6a. The FE model follows a similar trend with crack extension slowing at the locations of second and third rows of pins (9 mm and 15 mm, respectively), effectively capturing the experimental observations (Figure 6a). Further pin failure analysis was conducted to determine the pin failure modes and their sequence, as illustrated in Figure 6b, using the spring reaction force fraction vs. displacement plot.

Pin failure analysis was conducted on pin 1 and pin 2, positioned in the middle of the first and second rows, respectively. Pin 1 was located at the center of the initial 6 mm disbond, while pin 2 resided within the intact adhesive layer. FE models for disbond-TTR specimens indicated that pin 1 failed under shear force before mixed-mode cohesive disbonding was initiated. Following cohesive zone failure, pin 2 sheared in mode II, and pin 1 failed in mode I due to pin pullout. Pin 1 eventually pulled out, dominated by shear mode, but no crack propagated as the crack had already reached pin 2. Subsequently, the pullout of the first row with subsequent crack growth resulted in an increase in crack surface opening near pin 2, leading to the pin 2 failure in mode I. Although the spring failure progression observed in the FE model showed extended displacement (6–8 mm) compared to the experimental observation, where pin progressive failure occurred in the region of 5–5.5 mm, FEA is still able to capture the progressive pin failure mode behavior. A complete summary of the PTTR performance from the experimental test and the FE model performance is shown in Table 2. Experimental work provides direct observation of crack propagation, while FEA offers a deeper understanding of pin progressive failure, which is challenging to observe experimentally. FEA results align with experimental findings by replicating crack patterns and failure modes, enhancing our understanding of disbonding behavior in skin–stringer configurations. This synergy refines pin parameters, aiding in the design of more reliable composite structures.

## 5. Conclusions

The proposed PTTR of skin–stringer laminated composites allowed for a 3D reinforced adhesively bonded composite, with improved damage tolerance and mechanical behavior under flexural loads. PTTR was enabled by micro-drilling of submillimeter through-thickness circular holes and bonding fibrous carbon rods into the cured host laminate via low-viscosity resin. The reinforcement of cured composites ensures the versatility of the proposed method and ease of implementation, which can be automated using robotic microdrilling [28]. The investigation of crack resistance of the PTTR technique was characterized on pristine skin–stringer specimens and with specimens that had an initial edge disbond. Experimental observations showed that the proposed technique of TTR provided a 41% and 62% increase in crack initiation capability under mixed-mode loads for pristine and disbonded specimens, respectively, enhancing the reliability of adhesively bonded, stiffened composite structures. This study demonstrated an increase in crack resistance via the PTTR technique, which could be further enhanced with increases in the thickness-to-rod diameter ratio of laminates. The quasi-static bending of the skin–stringer configuration is an important test to characterize progressive disbonding failure; however, additional testing setups and loading conditions would be necessary to fully capture the performance of PTTR. This includes fatigue loading, which is more representative of real-world loading conditions, while additional testing configurations could include tensile-bending, and seven-point flexural testing [43].

The proposed approach to FEA modeling of pin–spring demonstrated the ability to efficiently represent the discrete arrays of pins within the laminate, while the inclusion of individual rows of holes into the FEA model captured the presence of TTR on the overall laminate mechanical behavior. The proposed FEA modeling strategy can be readily incorporated with the existing progressive failure analysis techniques to capture the overall structural response of PTTR laminate, allowing researchers to consider the knockdowns on stiffness, adhesive strength, and crack extension scenarios. Furthermore, the TTR pin–spring modeling approach was shown to accurately capture different pin failure modes, allowing us to capture the role of pin aspect ratio on the effective mechanical properties of PTTR. This modeling approach can be highly beneficial in predicting mechanical response in damage-tolerant structures by designing various PTTR arrangements, and locally introducing through-thickness reinforcements where significant interlaminar stresses are expected, thus enabling an improved structural behavior.

## Figures and Tables

**Table 1:** Material properties used for FEM.

Symbol	Material Constants (Units)	Magnitude
	Skin and stringer laminate engineering constants	
*E*_1_	Longitudinal Young’s modulus (GPa)	143
*E*_2_, *E*_3_	Transverse Young’s modulus (GPa)	8
*v*_12_	Poisson coefficient	0.32
*G*_12_, *G*_13_	Shear modulus (GPa)	3.1
*G*_23_	Shear modulus (GPa)	2.7
Pristine cohesive element properties		
*K*_1_	Normal penalty stiffnesses (N/mm)	3333
*K*_2_, *K*_3_	Shear penalty stiffnesses (N/mm)	500
*Tc*	Normal interfacial strength (MPa)	57
*S*_12_, *S*_13_	Shear interfacial strength (MPa)	38
$G_{I}^{C}$	Energy rate per unit area in normal direction (mJ/mm^2^)	0.32
$G_{II,\;\;\;}^{C}G_{III}^{C}$	Energy rate per unit area in shear direction (mJ/mm^2^)	0.80

**Table 2:** Summary of skin–stringer three-point bending and FE model prediction results.

	Experimental		FE Model	
	Stiffness (N/mm)	Failure Load (N)	Stiffness (N/mm)	Failure Load (N)
Pristine	159.25 ± 3.86	854.66 ± 13.63	166.28	839.32
Pristine-TTR	148.87 ± 4.05	987.7 ± 17.40 (15.5% improvement)	167.40	1009.77
Disbond	136.36 ± 6.52	564.47 ± 12.83	139.68	592.41
Disbond-TTR	149.66 ± 5.84	682.15 ± 39.92 (20.9% improvement)	145.94	810.87

## Data Availability

The original contributions presented in the study are included in the article, further inquiries can be directed to the corresponding author.
